# Nurse shift patterns, staffing and their association with perceived workload: Sequence analysis of multicentre data

**DOI:** 10.1016/j.ijnsa.2025.100420

**Published:** 2025-09-12

**Authors:** Tania Martins, Sarah N. Musy, Michael Simon

**Affiliations:** Nursing Science, Department of Public Health, Faculty of Medicine, University of Basel, Basel, Switzerland

**Keywords:** Nursing, Shift work schedule, Overtime, Workload, NASA-TLX

## Abstract

**Introduction:**

Although shift work is inevitable in hospitals, some shift patterns and staffing levels are suggested to influence nurses’ workload more than others, which in turn can impact nurses’ health, quality of care, and patient safety. Despite the importance of workload in nursing practice, studies focusing on nurses’ work schedules, staffing levels and perceived workload are rare. The aims of this study were to describe key characteristics of nurses’ shift work patterns in acute care hospitals, and to investigate the association of shift work patterns and staffing levels with perceived workload.

**Methods:**

This was a secondary analysis of an observational, cross-sectional, multicentre study conducted in 26 acute care hospitals in Switzerland. Registered nurses from 158 units completed the survey, covering questions about nurse staffing, the work environment and quality of care. We used sequenced data analysis to visualise nurses’ work schedules over the last seven days and identify shift characteristics and transitions. Clustering using Optimal Matching allowed us to group nurses with similar shift sequences and identify shift patterns. An observed-over-expected patient-to-nurse ratio (including patient acuity measures) was computed to assess staffing exposure. Perceived workload was measured with the NASA-Task Load Index instrument. A linear-mixed model was used to explore the association between identified shift work patterns, staffing and perceived workload.

**Results:**

We analysed surveys of 1962 registered nurses. The sequence analysis identified 732 different sequences resulting in three clusters of different shift patterns. Backward rotations, quick returns and working more than five consecutive days were rare. Workload perception was on average 66.5 points (possible range 6–120). Low staffing (β=3.1, 95 % CI [0.5–5.6]), overtime in the last shift (β=8.8, 95 % CI [7.2–10.4]), higher percentage of days worked overtime in the previous seven days (β=3.9, 95 % CI [1.3–6.3]), number of days worked (β=6.4, 95 % CI [2.5–10.1]), last shift worked being a day shift (β=3.8, 95 % CI [1.8–5.8]), and longer shift length (β=1.4, 95 % CI [0.5–2.2]) were associated with higher perceived workload.

**Conclusions:**

This study highlights the contribution of staffing and scheduling practices to nurses’ perceived workload. To reduce nurses’ perceived workload and improve healthcare performance—as previous research suggests—staffing and scheduling decisions must be increasingly prioritized by decision makers. The results suggest that avoiding or reducing e.g., overtime, reducing shift length and increasing staffing may be effective first strategies to reduce the perceived workload. Further research would benefit from analysing shift patterns using electronic rosters, real-time staffing measures, and repeated assessment of nurses’ workload perceptions.

**Social media abstract:**

Subjective nurse workload is influenced by shift patterns and staffing. Less overtime, shorter shifts, and better staffing may reduce workload.

**What is already known**

• High nurse workload is considered a major threat to quality of care, as it can affect nurses’ health, work performance and patient safety.

• Subjective workload measures provide a valuable, personalized, and context-specific assessment of the level of work demands.

• What influences nurses’ perception of their overall workload remain relatively unknown.

Alt-text: Unlabelled box

**What this paper adds**

• Using sequence analysis of 8292 shifts, we identified the types of shifts nurses worked, along with their frequencies, length, transitions and quick returns.

• Unfavourable scheduling practices, such as backward rotations, quick returns, 12-hour shifts, and working more than five consecutive days, were rare.

• Lower staffing levels, working a day shift, overtime and longer shifts were statistically significant associated with higher perceived workload.

Alt-text: Unlabelled box

## 1 Introduction

High workloads of nurses can have detrimental effects on patients, nurses and the healthcare system as a whole, being considered a major threat to quality of care (Van Bogaert et al., 2017), patient safety (Thomas-Hawkins et al., 2020), workforce performance (Montgomery et al., 2015), and nurses’ health, such as increased fatigue, stress and burnout (Cramer and Hunter, 2019). Increased workloads, unattractive working conditions, and poor work environments contribute to nurses' intention to leave, exacerbating the nursing shortage (Holland et al., 2019). Thus, it is increasingly important to develop strategies to reduce workload to retain nurses and ensure the delivery of safe and high-quality care.

Nurse workload is a multidimensional construct that refers to the performance required to carry out nursing activities (Morris et al., 2007). Workload can be described through objective and subjective measures. A widely used objective measure of workload is the patient-to-nurse ratio, which refers to the number of patients assigned to a nurse during a shift (Aiken et al., 2002). Although frequently used, this indicator disregards patient acuity and skill-mix. Furthermore, due to the high complexity and intensity of patient care or non-direct care activities (e.g., education of other nurses/students, administrative work), nurses can experience high workload even with a lower number of patients to care for.

Subjective workload measures have gained increasing interest in healthcare research as they provide a valuable, personalized, and context-specific assessment of the level of work demands (Griffiths et al., 2020). Workload perceptions consider individual characteristics of the nurse (e.g., skill levels/expertise, personal attributes), the interaction with work environment factors (e.g., working hours, available staff, complexity of care), and broader work conditions, such as support, teamwork and involvement in non-direct care activities (Alghamdi, 2016).

In nursing, shifts typically range from 8 to 12 h shifts, with the most common approaches dividing the day into either two 12-hour (morning and night) or three 8-hour shifts (morning, evening, and night) (Banakhar, 2017). Shift work can be divided into two broad types: 1) fixed shift systems, i.e., working the same shift for a prolonged time, and 2) rotating shift systems, i.e., changing shifts regularly. The rotating shift system can be divided in slowly rotating shift system (over a period of 2–4 weeks) and in rapidly rotating shift systems (changing every 3–5 days) (Chang et al., 2018). Working in rotating shifts has been associated with burnout (Wisetborisut et al., 2014) and occupational injuries (Hosseinabadi et al., 2019). In rotating shifts, a forward rotation is when shifts move from a morning to an evening and then to a night shift (clockwise). The inverse is called a backward rotation. Forward rotation aligns better with the endogenous circadian rhythm and is, therefore, suggested to reduce its disruption (Knauth, 1993), whereas backward rotation has been associated with poorer sleep quality (Shon et al., 2016). When in a backward rotation a shift change occurs within 11 h or less (e.g., evening to morning shift), it’s called a quick return (Vedaa et al., 2016). Quick returns can lead to severe sleepiness (Karhula et al., 2013), as well as higher risk of absence due to sickness (Vedaa et al., 2017).

Shift work can impair cognitive functioning (Bufano et al., 2024). Fixed evening shifts have been associated with increased sickness absence (Merkus et al., 2012), while night work has been shown to disrupt the circadian rhythm, impair sleep and work-life balance (Caruso, 2014), and reduce decision-making competence (Peng et al., 2022), among other effects. Without sleep, our cognitive and emotional abilities are disturbed, thus being a fundamental aspect to maintaining health and working safely (Krause et al., 2017; Turchi et al., 2019).

Nurses can also work beyond the scheduled working hours, i.e., work overtime. Overtime is more common in jobs with high demands (van der Hulst et al., 2006) and has been associated with higher perceived workload (Sturm et al., 2019), increased sleepiness, fatigue, stress (Vedaa et al., 2016), and increased rates of work-related injuries and illnesses (de Castro et al., 2010), and burnout (Watanabe and Yamauchi, 2018).

To date, no studies which describe the nurses’ work schedules, exploring all the different patterns were found. The evidence on the impact of the different shift patterns on workload perception is scarce, and studies have mostly been conducted in intensive care units (Mohamadi et al., 2019; Tubbs-Cooley et al., 2018), emergency departments (Bazazan et al., 2019), or perianesthesia (Young et al., 2008), and have mainly focused on specific domains of workload (e.g., mental workload) and not on the overall workload. Little and inconclusive evidence exists also for the association of objective workload measures (e.g., patient-to-nurse ratio) and nurses’ perceived workload (subjective measure) (Ivziku et al., 2022; Wang et al., 2021). Prior studies in healthcare have used sequence analysis, for example, to explore patients’ care trajectories, including health states, outcomes, care processes, and social, biological or psychological characteristics (Mathew et al., 2024). Given its potential to identify groups of individuals with similar patterns or trajectories across sequences of events (in our case, shift types), sequence analysis offers a suitable approach for examining shift work patterns.

This study addresses these gaps by providing, for the first time, a comprehensive analysis of nurses’ work schedules, staffing levels, and their association with overall perceived workload across acute care hospitals. Specifically, the aims of this study were to: 1) describe key characteristics of nurse shift work patterns, 2) assess staffing levels in acute care hospitals, and 3) investigate the association of shift work patterns and staffing with workload perception. We hypothesise that how nurses perceive the workload on the last shift (i.e., the most recent shift) might be influenced by the characteristics of that shift, as well as by staffing levels on the last shift and the shift patterns of the previous seven days.

Describing the shift work patterns, and staffing levels that are associated with high workload perception could help healthcare organisations and policymakers identify sustainable staffing, work scheduling, and workload management strategies.

## 2 Methods

### 2.1 Study design and setting

We conducted a secondary analysis of the observational, cross-sectional, multicentre Matching Registered Nurses services with changing care demands (Match^RN^) study. The aim of the Match^RN^ study was to examine changes in patient safety indicators and in the processes and structures of nursing care since the introduction of Swiss Diagnosis-Related Groups (Bachnick et al., 2017). We collected data through questionnaires in acute care hospitals in Switzerland’s three language regions (German, French and Italian), representing a range of acute care hospitals, from small rural hospitals to large university hospitals. In this study, we analysed data from the second wave in 2018. The same hospitals that participated in the Swiss arm of the Nurse Forecasting in Europe (RN4CAST) study were invited to participate in the Match^RN^ study. The RN4CAST study aimed to improve forecasting models by including how nurses’ work environments and qualifications impact nurse and patient outcomes. Data were collected between July 2010 and December 2011 in twelve European countries (Sermeus et al., 2011). Eligible hospitals had at least 60 acute care beds, employed at least 50 registered nurses, and were selected though quota sampling to reflect variation by language region and canton (Ausserhofer et al., 2012).

More detailed information on the Match^RN^ study can be found elsewhere (Bachnick et al., 2017).

According to current labour law in Switzerland, one quick return per week, 50 h (or more in exceptional cases) and working seven consecutive days per week are allowed (Schweizerische Eidgenossenschaft, 2023).

### 2.2 Participants

Registered Nurses (RNs) across 26 Swiss acute care hospitals and 158 units (medical, surgical, mixed medical-surgical, and other), providing direct patient care, completed the questionnaires covering questions about nurse staffing, work environment, and quality of care. A total of 2463 registered nurses participated in the study, with an average response rate of 75.4 % per unit (range: 32 %–100 %). Non-registered nurses (e.g., certified nurses, nurse assistants) were excluded. Further information on the data collection approach can be found in the Match^RN^ sudy protocol (Bachnick et al., 2017).

### 2.3 Variables and measurements

#### 2.3.1 Outcome variable

To measure nurses perceived workload during their last shift, we used the National Aeronautics and Space Administration Task Load Index (NASA-TLX) (Hart and Staveland, 1988), as it is one of the most reliable and valid instruments for measuring workload perception (Hoonakker et al., 2011). The instrument measures six dimensions (mental demands, physical demands, temporal demands, performance, effort, and frustration) with ratings on a twenty-point scale ranging from 1=low, to 20=high (or for performance, from poor to good). Originally, the overall workload measured by the NASA-TLX is based on a weighting sum procedure, but researchers often use the raw test scores, where the ratings from each dimension are added together unweighted, to create an estimate of overall workload. The elimination of the weighting process is the most common modification made to the NASA-TLX, as it is simpler to apply and studies have found no disadvantages in terms of sensitivity (Hart, 2006). Our overall perceived workload measure resulted also from the unweighted sum score of the six NASA-TLX items, with possible scores ranging from 6 to 120, with higher scores representing higher perceived workload. When testing the reliability of the NASA-TLX, Cronbach's alpha in our sample was 0.73 (95 % CI: 0.72–0.76).

#### 2.3.2 Explanatory variables

##### 2.3.2.1 Shift work patterns

Information on the shift work system was assessed using a single item: ‘Which answer most likely applies to your service plan/shift in this hospital? I mainly work…’. The following answer options were possible: ‘morning’, ‘evening’, ‘morning and evening without night’, ‘morning, evening and night’, ‘night’ and ‘I always work the same shift (e.g., 8am until 4pm)’. Nurses who answered ‘I always work the same shift’ were considered to work mainly in a fixed shift system, while the others were considered to work mainly in a rotating shift system.

A seven-day timetable (Monday-Sunday) was provided for nurses to fill in what time (hours: minutes) they started and finished work (regular and overtime) each day, and when they were off during the previous week. This item allowed us to assess: a) the number of days worked in the last seven days, b) the number of consecutive days worked and days off, c) the type of shift worked (morning, evening, night), d) the duration of the shift, e) on which days nurses worked overtime and for how long, f) the direction of the rotation of shift changes (e.g., morning to evening shift = forward rotation, evening to morning shift = backward rotation), and g) the presence of quick returns (change from evening to morning shift within 11 h or less).

##### 2.3.2.2 Staffing levels

To assess the staffing levels, we used items from the RN4CAST study (Sermeus et al., 2011), after translation and adaptation to the Swiss context. RNs were asked, with single items referring to the last shift, about: 1) how many patients they were responsible for; 2) how many patients were in total on the unit; and 3) counting themselves how many RNs were providing direct patient care on the unit. This allowed us to describe the patient-to-nurse ratio. One item asked how many other nursing care staff were providing direct patient care in order to assess the skill-mix. To assess patient acuity, RNs were asked,of all the patients they were responsible for, how many required assistance with all activities of daily living, how many required hourly or more frequent monitoring or treatment, and how many were discharged or admitted.

Shift length and overtime were assessed using single items. RNs were asked to provide the number of hours they worked on their last shift in this hospital and the number of overtime hours they have worked. RNs were asked to select which shift type best describes their last shift, with the following answer options: morning, evening and night, which were dichotomised into ‘day’ (morning and evening) and ‘night’ shifts for the analysis.

#### 2.3.3 Socio-demographic characteristics

For this analysis we used socio-demographic characteristics such as sex (female/male), age (years), nursing education (master’s degree, bachelor’s degree, vocational training and other) and employment status (full-time/part-time). As work experience was highly correlated with age in our sample (Pearson’s *r*= 0.88), and had more missing values, we included only age in the analysis. In addition, we used information on the unit type (surgical, medical, mixed, and others).

### 2.4 Statistical analysis

Depending on the variable, between 0.2 % and 4.6 % of data was missing, we therefore decided to use listwise deletion for cases with missing data. Plausibility and consistency of the data was checked, and inconsistent values were deleted and treated as missing data.

We described the socio-demographic characteristics of nurses, the shift work patterns, and staffing levels, calculating frequencies, means and their standard deviations (SD), and medians with interquartile ranges (IQR).

#### 2.4.1 Shift work patterns

For the first aim, based on the reported starting times, we identified typical times and labelled them into three shift types: ‘morning’, ‘evening’ and ‘night’. Starting times before 11am were coded as ‘morning’, between 11am and 5pm as ‘evening’ and after 5pm as ‘night’ shifts. Night shifts were considered to be a shift of the day where the night begins. Working hours were ascribed to the respective shift and not to the day. When nurses were off duty, shift type was coded as ‘off’. The number of days worked were transformed into the proportion of days worked in the week, and the number of days with overtime was converted into the proportion of the days worked (overtime days divided by the total number of days worked). Regarding the shift length system, we categorized nurses working in 12-hour and in <12-hour shift systems.

As a next step, we conducted a sequence analysis of the different labelled shift types (off, morning, evening and night)*.* A sequence is “*an ordered list of states (*e.g., *employed/ unemployed) or events (*e.g., *marriage, having a child*)” (Gabadinho et al., 2010, p. 10). Sequence analysis allows to describe sequences, i.e., shifts present, their frequencies and the most frequent shift changes. It allowed us to identify the number of mornings, evenings and nights worked, as well as the transitions between each type o shift, and to identify forward and backward rotations, and quick returns. If at least one change in shifts in the past seven days occurred within 11 h or less, we considered that the RNs had quick returns.

To identify shift patterns, we clustered our sequenced data to group nurses with similar shift sequences together into one group and separate nurses with dissimilar sequences into another group. Many sequence dissimilarity measures have been proposed in the literature, of which the most popular in the social sciences is the Optimal Matching (OM) distances. OM is a method of calculating the sequences with the smallest average absolute distance (the most similar ones) across all sequences (Gabadinho et al., 2010). We used OM with a constant insertion/deletion cost of 1 and a data-driven substitution-cost matrix based on observed transition rates in the sample. The resulting dissimilarity matrix was clustered using agglomerative hierarchical clustering (Ward’s method) (Ward Jr, 1963). We evaluated the optimal number of clusters with different statistical testing (silhouette coefficient, elbow, and gap statistic method), which suggested between three and four clusters. A three-cluster solution was selected as the more parsimonious and interpretable choice (see Supplementary Material Figure S1).

#### 2.4.2 Staffing levels

For the second aim, to assess staffing levels, we first developed a staffing model to describe a) the overall staffing level for each unit and b) the staffing levels experienced by individual nurses during the last shift. The staffing model predicts the number of patients a nurse cares for, which is equivalent to the patient-to-nurse ratio, based on several staffing characteristics. These characteristics include: 1) the number of RNs and the number of other nursing care staff (skill mix), 2) the total number of patients present in the unit during the last shift, 3) the shift type, 4) the three patient acuity measures and 5) the unit type. Patient acuity measures were transformed into percentages, considering the total number of patients for which RNs were responsible for. Last shift type worked was dichotomised into ‘day’ (morning or evening) and ‘night’ shift.

Second, we used a generalised mixed model of the Poisson family, which accounted for the dependence of observations with random effects for units (intraclass correlation coefficient (ICC) (1) 0.06) and hospitals (ICC (1) 0.12) (see formula in the Supplementary Material Figure S2 ). From this prediction model we extracted the conditional mean of the patient-to-nurse ratio by unit with the empirical Bayes estimate (Gu and Koenker, 2017). We considered this as the time-invariant unit-level staffing measure. This staffing model allowed us to identify the average staffing on the unit level, but also in a second step whether the individual staffing exposure on the last shift was below or above the typical staffing on the given unit. We therefore divided the observed patient-to-nurse ratio on the last shift by the expected patient-to-nurse ratio from the staffing model, an observed-over-expected estimator (O/E) (Musy et al., 2020).

Based on the O/E, we used the 0.15 and 0.85 quantiles to define staffing exposure at the individual level. This threshold was set based on a previous study using this estimator (Sharma et al., 2022). Values below 0.15 were considered as high staffing, between 0.15 and 0.85 as medium and above 0.85 as low staffing (more patients per nurse observed than it would be expected). Individual staffing exposure was categorised in low, medium and high. The 0.85 quantile was defined as ≥1.24, indicating that the observed number of patients each RN was responsible for was higher than expected (low staffing). For high staffing, the 0.15 quantile was defined as <0.74, indicating that the observed number of patients each RN was responsible for was lower than the expected number. For staffing at the unit level, higher values indicate that staffing was lower than the mean for that unit (i.e. higher variation between actual individual staffing and the unit’s conditional mean), given the prediction model used.

#### 2.4.3 Shift work patterns, staffing and workload perception

Finally, for the third aim, we assessed the association with shift patterns and staffing on short-term workload perception with a linear mixed-effects model. We used a mixed model because the ICC (1) from unconditional random intercept models with hospital (ICC (1) 0.09) and unit (ICC (1) 0.17) as random effects indicated dependence between individuals within units and hospitals (Snijders and Bosker, 2012). The prediction model included characteristics of the last shift, staffing at the individual level (categorised as low, medium, high), last shift type (day/night), number of hours worked and overtime in the last shift (dichotomised as <0.5h: no, ≥ 0.5: yes). As the explanatory shift pattern characteristics, we considered, the shift length system (<12 h, ≥12 h), proportion of days worked overtime in the last seven days, shift patterns (identified clusters) and presence of quick returns (yes/no) (see formula in the Supplementary Material Figure S3). To control for confounders, we included the proportion of days worked and age in the model as control variables. Variable inclusion was based on existing evidence regarding shift characteristics and factors influencing workload, as well as their availability in the dataset. Before running the models, we assessed multicollinearity between explanatory variables using the variance inflation factor (VIF) (i.e. VIF < 5) (Thompson et al., 2017). All VIF values were below 2, indicating no multicollinearity. Model selection was based on subject-matter knowledge, backward selection, the Akaike information criterion (AIC), and the Wald statistic (Snijders and Bosker, 2012). Model assumptions were assessed by examining normality of residuals, homoscedasticity, and influential observations. Residuals were approximately normally distributed, residual plots indicated homoscedasticity, and no influential observations were detected. Statistical significance was set at the *p* < .05 and unstandardized regression coefficients (β) and incidence rate ratio (IRR), 95 % confidence intervals (CI) and coefficient of multiple determination (R^2^) were reported. To explore potential selection bias due to missing data, we compared the excluded cases with those included in the analysis using independent samples *t*-tests for continuous variables and chi-square tests for categorical variables. All statistical analyses were performed using R, version 4.1.3 for Mac OS (R Core Team, 2022) and the R packages *lme4* (Bates et al., 2015), *tramineR* (Gabadinho et al., 2011), *cluster* (Maechler et al., 2021), *psych* (Revelle, 2022) and *tidyverse* (Wickham et al., 2019). Data are available at https://doi.org/10.5281/zenodo.15248072.

### 2.5 Ethical considerations

The Match^RN^ study was approved by the hospital management and received exempt status by the respective ethics committee (Ref. Nr. EKNZ UBE 15/59). The nurse questionnaire included a cover letter that explained the objective of the study, assured the confidentiality of the data collection process and data protection. By completing and sending the survey RNs gave their consent to participate. Further information can be found in the Match^RN^ study protocol (Bachnick et al., 2017).

## 3 Results

In the Match^RN^ 2018 study, 2463 RNs participated. Our sample consists of 1962 RNs (79.6 % of the initial sample). A flow chart with the excluded cases due to missing data is displayed in Supplementary Material Figure S4. We did not find statistically significant differences between the study and the excluded sample, except for age and perceived workload (Supplementary Material Table S1).

### 3.1 Descriptive overview

RNs had a mean age of 35.5 years (SD 10.7), were mostly female (88.8 %, *n* = 1743), and 44.2 % (*n* = 867) worked full-time. Nurses’ socio-demographic characteristics are listed in Table 1.

**Table 1:** Socio-demographic characteristics of the RNs (*n* = 1962).

Variables	n ( %)
Sex	
Female	1743 (88.8)
Male	219 (11.2)
Employment status	
Full-time	867 (44.2)
Part-time	1095 (55.8)
Nursing Education	
Master’s degree	36 (1.8)
Bachelor’s degree	456 (23.2)
Vocational training	1394 (71.1)
Other	76 (3.9)
Unit type	
Medical	668 (34.0)
Surgical	622 (31.7)
Mixed	331 (16.9)
Others	341 (17.4)

*Note*. n, number.

On their last shift, most (78.6 %, *n* = 1542) of the RNs were working a day shift, a mean shift length of 8.9 h (SD 1.2), and were responsible for a mean of 7.5 patients (SD 4.1). RNs reported that a median of 25.0 % (IQR 0.0–50.0) of the patients they cared for during day shifts required hourly or more frequent monitoring or treatment, compared to 16.0 % (IQR 0.0–33.3) during night shifts. Last shift characteristics can be found in Table 2.

**Table 2:** Last shift’s characteristics by day (morning and evening shifts) and night shift (*n* = 1962).

Variables	Day shift *n* = 1542			Night shift *n* = 420		
	Mean (SD)	Median (IQR)	Range	Mean (SD)	Median (IQR)	Range
Shift length (h)	8.8 (1.1)	8.5 (8.0–9.0)	4.0–14.0	9.3 (1.3)	9.0 (8.5–9.0)	5.0–15.0
Patient-to-nurse-ratio	6.3 (2.7)	6.0 (5.0–8.0)	1.0–26.0	11.7 (5.6)	11.0 (8.0–14.0)	1.0–40.0
N° of patients in the unit	19.4 (7.3)	19.0 (15.0–23.0)	1.0–53.0	18.9 (7.8)	19.0 (13.0–23.0)	1.0–53.0
Percentage RNs over all nursing care staff	66.1 (17.8)	66.7 (50.0–75.0)	25.0–100.0	85.5 (21.2)	100.0 (66.7–100.0)	33.3–100.0
Patient acuity ( %) of patients responsible for who…						
…required assistance with all ADL’s ( %)	51.3 (30.1)	50.0 (28.6–75.0)	0.0–100.0	45.1 (28.2)	41.7 (23.8–65.6)	0.0–100.0
…required hourly or more monitoring/ treatments ( %)	30.5 (28.9)	25.0 (0.0–50.0)	0.0–100.0	22.9 (24.8)	16.0 (0.0–33.3)	0.0–100.0
…were admitted or discharged ( %)	19.4 (22.4)	14.3 (0.0–30.0)	0.0–100.0	7.4 (13.0)	0.0 (0.0–10.0)	0.0–100.0

*Note*. n, number; IQR, interquartile range; SD, standard deviation; RNs, registered nurses; ADL’s, activities of daily living.

Of the RNs working a day shift, 39.3 % (*n* = 606) worked half-our or more overtime, while 23.1 % (*n* = 97) of those working a night shift worked half-our or more overtime. Over all shifts, half-hour or more overtime in the last shift was reported by 35.8 % (*n* = 703) RNs. Considering all overtime lengths (also less than half-hour), 58.4 % (*n* = 1146) reported overtime in their last shift. Additionally, 30.2 % (*n* = 592) RNs worked overtime (all durations considered) during the previous seven days. Supplementary Material Table S2 shows the characteristics of overtime worked.

Concerning perceived workload, the raw sum score of the six perceived workload subscales showed an overall perceived workload mean sum score of 66.5 (SD 17.2) (possible range: 6–120; range in our sample: 10–116) points.

### 3.2 Shift work patterns

Of the seven analysed days, data on 8292 shifts were available. The majority of the RNs, 74.8 % (*n* = 1368), had a rotating shift system and 7.1 % (*n* = 140) worked in a 12-hour shift system. Table 3 provides an overview of the previous seven days shift patterns’ characteristics.

**Table 3:** Shift work patterns’ characteristics (*n* = 1962).

Characteristics	n ( %)	Mean (SD)	Median (IQR)	Range
**N° of days worked**		4.2 (1.3)	4.0 (3.0–5.0)	1.0–7.0
>5	332 (16.9)			
5	610 (31.1)			
<5	1020 (52.0)			
**N° of consecutive days**		3.2 (1.3)	3.0 (2.0–4.0)	1.0–7.0
>5	70 (3.5)			
5	273 (13.9)			
<5	1619 (82.6)			
**Shift types (days)**				
Morning		2.2 (1.8)	2.0 (0.0–4.0)	0.0–7.0
Evening		1.3 (1.5)	1.0 (0.0–2.0)	0.0–7.0
Night		0.8 (1.3)	0.0 (0.0–1.0)	0.0–7.0

*Note.* SD, standard deviation; IQR, interquartile range.

RNs enrolled in 12-hour shift systems had on average 3.3 days off (SD 1.1), while RNs on shift systems less then 12 h had on average 2.7 days off (SD 1.3). Working more than five consecutive days was present in 3.5 % (*n* = 70) of our sample.

The sequence analysis identified 732 different sequences. The most frequent sequence observed was five morning shifts with two days off, however only 5.1 % (*n* = 100) of all nurses worked in this pattern. Concerning the direction of rotation, backward rotation was found in 4.1 % (*n* = 80) of the work schedules and 3.6 % (*n* = 70) of the RNs had quick returns. Further details on the most frequent sequences and transitions can be found in Supplementary Material Figure S5 and Table S3.

**Fig. 1:**
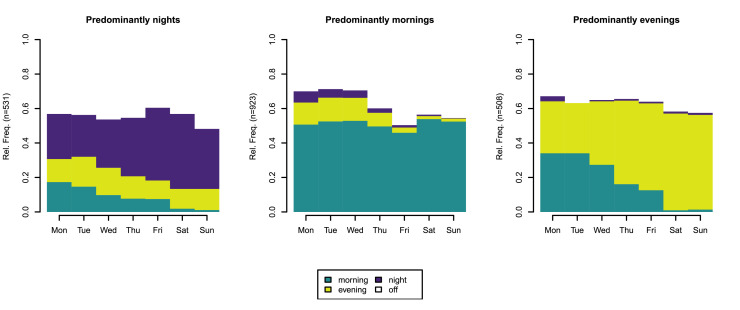
Identified shift patterns.

*Note.* Rel. Freq., relative frequency; n, number of RNs

Three different clusters of shift patterns were identified. The first cluster was characterized by predominantly night shifts, the second by predominantly mornings and the third by predominantly evenings (see Fig. 1).

All three shift types (morning, evening, night) in the time span of seven days were experienced by 5.2 % (*n* = 103) of the RNs. Most frequent sequences in each cluster can be found in Supplementary Material Figure S6 and Figure S7.

### 3.3 Staffing levels

Results from the staffing model—which aimed to identify average staffing at the unit level and determine whether individual staffing exposure was below or above the typical levels—showed that for a day shift in a medical unit with an average number of patients (19.4), an average percentage of RNs among all nursing care staff (66.1 %), and average patient acuity (51.3 % requiring assistance with all activities of daily living, 30.5 % requiring hourly or more monitoring/treatments, and 19.4 % admitted or discharged), the predicted average patient-to-nurse ratio was 6.2 (95 % CI [5.4–7.1]. For night shift, the patient-to-nurse ratio increased to 11.3 patients (95 % CI [9.8–13.0]. More details of the staffing model can be found in Supplementary Material Table S4.

### 3.4 Shift work patterns, staffing and workload perception

From our initial (full) perceived workload model (see Supplementary Material Table S5), the presence of quick returns and working in a 12-hour shift system were not statistically significant and did not explain any variation in perceived workload. The final model showed that when the last shift was a day shift, perceived workload increased on average by 3.8 points (95 % CI [1.8–5.8]) on a scale from 6 to 120, holding all other variables constant, compared to a night shift. Nurses who had a longer shift had a perceived higher workload of on average 1.4 points (95 % CI [0.5–2.2]). Although statistically significant, this increase is small given the range of the NASA-TLX scale, suggesting limited practical importance. For each additional day overtime, workload increased on average by 3.9 points (95 % CI [1.3–6.3]). Workload was on average 8.8 points (95 % CI [7.2–10.4]) higher when overtime was worked on the last shift compared to when no overtime was worked. A higher proportion of days worked was associated with a 6.4-point increase in workload score per percentage point (95 % CI [2.5–10.1]). The three identified shift patterns of the previous seven days showed no significant association with perceived workload of the last shift. Staffing at the individual/nurse level was independently associated with perceived workload. For nurses with low staffing (O/*E*≥ 1.24) the NASA-TLX score was on average 3.1 points (95 % CI [0.5–5.6]) higher and for nurses with medium staffing 2.1 points (95 % CI [0.0–4.1]) higher, compared with nurses with high staffing. Although statistically significant, this increase is of limited practical relevance given the range of the NASA-TLX scale. The conditional explained variance of the model was 27 %. The predictors included in the final linear mixed-effects model, with summaries of their regression coefficients can be found in Table 4.

**Table 4:** Results of the final adjusted perceived workload model.

Variables	Estimates (β)	95 % CI	*p*-value
Intercept	40.3	31.0–49.4	<0.001
Staffing individual level (reference category – O/E: high)			
O/E: low	3.1	0.5–5.6	0.019
O/E: medium	2.1	0.0–4.1	0.047
Last shift length (h)	1.4	0.5–2.2	0.001
Last shift type: day	3.8	1.8–5.8	<0.001
Cluster (shift patterns) (reference category: cluster; predominantly nights)			
Cluster: predominantly mornings^a^	0.2	–1.7–2.1	0.853
Cluster: predominantly evenings^a^	0.6	–1.5–2.7	0.575
Days worked overtime ( %)	3.9	1.3–6.3	0.002
Overtime last shift: yes	8.8	7.2–10.4	<0.001
Days worked ( %)	6.4	2.5–10.1	0.001
Age (years)	0.01	–0.05–0.1	0.644
**Random Effects**	**Variance (SD)**		
Unit	22.2 (SD 4.7)		
Hospital	17.6 (SD 4.2)		
R^2^ marginal: 0.138 / R^2^ conditional: 0.271 Number of observations: 1962; number of units: 158; number of hospitals: 26 Akaike’s information criterion (AIC): 16,295			

*Note.* Estimates are unstandardized; CI, confidence interval; O/E, observed-over-expected estimator; R^2^, coefficient of multiple determination; SD, standard deviation.

The results of the unadjusted perceived workload model can be found in Supplementary Material Table S6.

## 4 Discussion

We analysed data from 1962 registered nurses (RNs) across 26 acute care hospitals in Switzerland to describe shift work patterns, staffing, and their relationship with perceived workload. Our results suggest that RNs in Swiss hospitals report higher levels of perceived workload than those observed in previous research. The presence of backward rotations, quick returns, or more than five consecutive days was low (<5 %). Lower staffing levels, working a day shift, overtime, and longer shifts were statistically significantly associated with higher perceived workload. In addition, having worked overtime on more days in the previous seven days was associated with higher perceived workload. In contrast, staffing patterns such as working predominantly morning shifts were not significantly associated with perceived workload.

### 4.1 Shift work patterns and staffing levels

The first aim was to describe key characteristics of nurse shift work patterns in Swiss acute care hospitals. Working in rotating shift systems was the most prevalent system (74.8 %) found in our sample similarly to studies conducted in Iran and Spain where it was 76.3 % and 65.4 % respectively (Gómez-García et al., 2016; Hosseinabadi et al., 2019). The identified shift patterns suggest that nurses mostly worked one or two different shifts per week, and while the occurrence of all three shift types was rare, it was not absent suggesting the existence of rapidly rotating shift systems.

We identified fewer RNs who reported overtime the last seven days compared to a study conducted in the US (Olds and Clarke, 2010), where the overtime rate was two times higher (63 %). In our study, 35.8 % of the RNs reported working more than half an hour of overtime on the last shift, which is higher than the 27 % reported in a study conducted across 12 European countries (Griffiths et al., 2014). In the same study, 41 % of the RNs in Switzerland reported overtime in their last shift (including also durations of less than half-hour), which was less than the overtime reported in our study (58.4 %). This suggests that nurses might be working more overtime, potentially due to increasing workloads.

Quick returns, backward rotations, and more than five consecutive days of work were rare. The Swiss State Secretariat for Economic Affairs, in its recommendations for reducing employee burden when designing shift schedules, emphasizes avoiding quick returns, using backward rotations only in exceptional cases, and limiting schedules to a maximal of 5–6 consecutive shifts (State Secretariat for Economic Affairs, 2016). These recommendations, together with existing research demonstrating the negative impact of these scheduling practices on staff health, may help explain why such patterns were rare in our sample.

For the second aim, we examined staffing levels. In our sample, the reported mean patient-to-nurse ratio was 6.3 during day shifts and 11.7 during night shifts, resulting in an overall average of 9.0 patients per nurse. These findings are similar to those reported in a previous study conducted in England across 48 hospitals, where the mean ratio was 8.66 (Emmanuel et al., 2020).

Insufficient staffing is one of the top reasons for nurses leaving their jobs (Muir et al., 2024) and is also associated with higher burnout levels and increased job dissatisfaction (Shin et al., 2018). Higher RN staffing, in contrast, has been shown to reduce patient mortality (Dall'Ora et al., 2022). While, for example, California’s safe staffing law (AB-394) mandates a maximum ratio of five patients per nurse in adult medical and postoperative surgical units, no such regulation exists in Switzerland (Simon et al., 2020).

### 4.2 Shift work patterns, staffing and workload perception

Concerning our third aim, we found a perceived overall workload mean sum score of 66.5 (SD 17.2) points on the NASA-TLX scale, which seems relatively high compared with a meta-analytic review on studies conducted in the healthcare settings which showed a mean score of 45.0 (SD 16.0) points on the NASA-TLX (Hertzum, 2021). However, to date no threshold is defined to identify critical levels of perceived workload. While we found that day shift work was associated with higher perceived workload, a previous cross-sectional study conducted in five emergency departments in Iran showed that permanent night workers had higher levels of perceived workload (Bazazan et al., 2019). Although we found that night-shift work was associated with lower perceived workload, it is important to be aware of the cumulative effects on the circadian misalignment (Boivin et al., 2022), which have been suggested, for example, to lead to impairments in neurobehavioral performance (Magee et al., 2016), cognitive slowing (Chellappa et al., 2018), and increased insomnia and fatigue (Alqahtani et al., 2024). Patient acuity and overtime worked were higher on day shifts than night shifts, which may help explain why we found higher perceived workload on day shifts. Similarly to our results, a previous study conducted in a Lebanese acute care hospital, reported lower perceived workload during the night shifts compared to day shifts, which they attributed to lower patient demands and fewer processes at night, as most diagnostics, treatments and admissions occur during the day (Abed Al Ahad et al., 2022).

We could identify three different clusters concerning shift patterns. We did not find evidence of statistically significant relationship between the shift patterns and perceived workload. However, as the clusters refer to the last seven days worked, we have to consider that the seven days may not be enough to sufficiently capture all shift patterns. As 732 different sequences were identified, each cluster contains various shift sequences. Although the clusters differed in their proportions of morning, evening and night shifts, their mean workload scores were nearly identical, leaving little between-cluster variation to detect. Shift length and overtime in the last shift were associated with higher perceived workload, as was working overtime on more days in the last seven days. This suggests that previous working patterns may, to some extent, influence how nurses perceive workload on the last shift. Working overtime may disrupt nurses’ work-life-balance, increase their fatigue and affect their performance and consequently their workload perception.

Although prior studies have associated 12-hour shifts with negative outcomes for staff, such as increased fatigue, intention to leave and burnout (Clari et al., 2024), this shift length was not statistically significantly associated with workload in our sample. A possible explanation for this finding is that RNs in our setting may have adapted to this shift system. Evidence suggests that some RNs prefer 12-hour shifts as they consider them to allow, for example, more time to handle new admissions, complete tasks, avoid gaps in staff coverage, ease commuting, and improve work-life balance (Haller et al., 2020). Another possible explanation is that the NASA-TLX, as a measure of short-term workload perception, may not capture the long-term consequences of 12-hour shifts.

Staffing levels were significantly associated with nurses’ perceived workload in our model. Although Switzerland has a high number of nurses per capita (OECD/European Commission, 2024), Swiss hospitals, like those in other countries, are confronted with increasing care complexity due to rising multimorbidity (Obsan, 2025), higher patient volumes (Federal Office of Public Health, 2025) and other challenges. These factors may help explain nurses working overtime, experiencing low staffing, and reporting higher perceived workloads. Importantly, the data were collected before the COVID-19 pandemic, which may have further increased nurses’ workload and intention to leave (Xiao et al., 2025), and is likely to have exacerbated difficulties in recruiting and retaining RNs.

Despite the inevitability of shift work, some shift patterns and staffing levels may perpetuate more stress for employees than others (Montgomery et al., 2011). In previous studies, professionals exposed to poorly scheduled work hours showed degraded performance, impaired thought processes, increasing the changes to err and higher levels of missed care (Tubbs-Cooley et al., 2019), thus compromising the quality of care. The effect sizes of the factors associated with perceived workload ranged from 1.4 to 8.8 on the NASA-TLX scale (scoring range 6–120), suggesting that their practical implications range from negligible to moderate.

Our study supports the notion that perceived workload is influenced by factors within the control of the organisations. As stated in a previous study (Tubbs-Cooley et al., 2018), how nurses experience workload is likely to be a strong additional predictor of patient and nurse outcomes compared to objective measures alone. Measuring nurses’ workload perception and identifying the drivers of high workloads enables the development of targeted interventions to reduce perceived workload and, consequently, burnout, fatigue, intention to leave, and errors. Scheduling practices and staffing levels are two examples of such drivers. A previous study indicated that subjective workload ratings were more consistently linked to care reliability (e.g., lower levels of missed care) than objective measures, suggesting that the direct influence of staffing ratios on care reliability may be overestimated (Tubbs-Cooley et al., 2019). Systematically monitoring how RNs perceive their workload alongside objective measures could provide insight into how each nurse experiences work demands including cognitive, physical, and time pressures. Incorporating these assessments, for example through digital tools, into daily practice could provide real-time information on experienced workload and allow for prompt adjustments in staffing and workload, helping to prevent negative outcomes for both patients and nurses.

### 4.3 Limitations

Although we were able to identify certain shift patterns characteristics and factors that could explain some of the variance in perceived workload, and analyse the association of objective and subjective workload measures, there are important limitations to consider.

The cross-sectional design allows no inference of causal relationships between the shift work patterns, staffing levels and perceived workload. Our sample consisted of a convenience sample, which may lead to a biased sample, and all variables were self-reported, which could lead to response bias. These factors may limit the generalisability of the findings, and therefore we recommend replication in other hospitals using a random sample.

As the study was conducted before the COVID-19 pandemic, comparisons with more recent studies conducted during and after the pandemic might be misleading, as workload increased and shift patterns and staffing levels were adapted to the context. Our model explains only 27 % of the variance in perceived workload, suggesting that other factors, such as individual characteristics, training, job resources or work-life balance likely influence the workload perception.

A major limitation is that we analysed shift patterns over just one week, which may not be representative of nurses’ typical work schedules. Another limitation is the possible inacurancy of the self-reported data on the number of patients, RNs on the units, and patient acuity measures, as participants may have been unable to precisely register staffing numbers while simultaneously providing care. Finally, perceived workload data were provided only for the last shift worked. Further research is thus needed, analysing shift patterns over a longer time, using longitudinal data, electronic rosters, and real-time staffing measures. Electronic rosters could be linked to digital platforms that would collect real-time workload measures (e.g., NASA-TLX) enabling workload ratings to be automatically matched with staffing data. Repeated measures of nurses' workload perceptions, along with the analysis of the factors contributing to high workloads, could inform managers about potential measures to reduce nurses' workload and improve both nurses and patient outcomes.

## 5 Conclusions

Nurses’ perceived workload in Swiss hospitals was relatively high. Quick returns, backward rotations, 12-hour shift system, and working more than five consecutive days were rare. The study highlights the contribution of staffing levels and scheduling practices on nurses’ workload perception. To reduce nurses’ high workload perception, staffing and scheduling decisions must be increasingly focused by decision makers. Reducing factors such as overtime and shift length, and increasing staffing levels, may be effective first strategies. Since other factors also influence workload perception, regularly monitoring scheduling practices, staffing levels, and perceived workload is indispensable to maintaining an optimal workload.

## Funding

This work was supported by the participating hospitals.

## CRediT authorship contribution statement

**Tania Martins:** Writing – review & editing, Writing – original draft, Methodology, Formal analysis, Conceptualization. **Sarah N. Musy:** Writing – review & editing, Methodology. **Michael Simon:** Writing – review & editing, Supervision, Methodology, Funding acquisition, Conceptualization.

## Declaration of competing interest

The authors declare that they have no known competing financial interests or personal relationships that could have appeared to influence the work reported in this paper.

## Data Availability

Data analysed in this study are available at https://doi.org/10.5281/zenodo.15248072.

## Appendix Supplementary materials

Image, application 1
