# Supplementary material for: Nurse shift patterns, staffing and their association with perceived workload: Sequence analysis of multicentre data
Source: Int J Nurs Stud Adv. 2025 Sep 12;9:100420. doi: 10.1016/j.ijnsa.2025.100420 (PMC12538485; doi:10.1016/j.ijnsa.2025.100420)
Supplement: Supplementary file 1 [file mmc1.zip › mmc1.zip!/Suppl_Material /Nurse_shift_workload_Supp_TableS6.docx]

**Table S6**

*Results of the final unadjusted perceived workload model (n=1,962)*

| **Variables** | **Estimates (β)** | **95% CI** | ***p*-value** |
| --- | --- | --- | --- |
| Intercept | 44.8 | 36.4–53.1 | <0.001 |
| Staffing individual level (reference category – O/E: high) |  |  |  |
| O/E: low | 3.1 | 0.5–5.6 | 0.018 |
| O/E: medium | 2.1 | 0.0–4.1 | 0.047 |
| Last shift length (h) | 1.3 | 0.5–2.2 | 0.002 |
| Last shift type: day | 3.6 | 1.6–5.6 | <0.001 |
| Cluster (shift patterns) (reference category: cluster; predominantly nights) |  |  |  |
| Cluster: predominantly mornings^a^ | 0.7 | –1.1–2.6 | 0.434 |
| Cluster: predominantly evenings^a^ | 1.2 | –0.9–3.2 | 0.272 |
| Days worked overtime (%) | 3.7 | 1.1–6.1 | 0.004 |
| Overtime last shift: yes | 9.0 | 7.3–10.6 | <0.001 |
| **Random Effects** | **Variance (SD)** |  |  |
| Unit | 22.4 (SD 4.7) |  |  |
| Hospital | 18.1 (SD 44.2) |  |  |
| R^2^ marginal: 0.134/ R^2^ conditional: 0.269  Number of observations: 1,962; number of units: 158; number of hospitals: 26  Akaike’s information criterion (AIC): 16301 | | | |

*Note.* Estimates are unstandardized; CI, confidence interval; O/E, observed-over-expected estimator; R^2^, coefficient of multiple determination; SD, standard deviation.
