# Supplementary material for: Nurse shift patterns, staffing and their association with perceived workload: Sequence analysis of multicentre data
Source: Int J Nurs Stud Adv. 2025 Sep 12;9:100420. doi: 10.1016/j.ijnsa.2025.100420 (PMC12538485; doi:10.1016/j.ijnsa.2025.100420)
Supplement: Supplementary file 1 [file mmc1.zip › mmc1.zip!/Suppl_Material /Nurse_shift_workload_Supp_FigureS1.pdf]

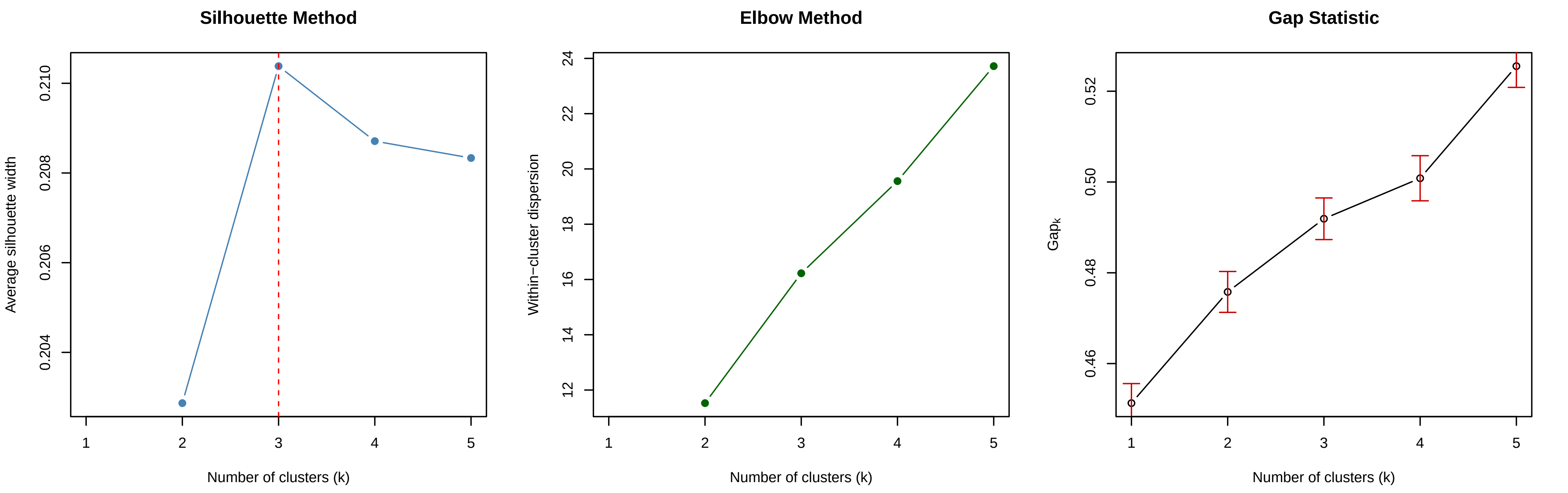
Silhouette Method
Average silhouette width
0.204
0.206
0.208
0.210
Number of clusters (k)
1
2
3
4
5
Elbow Method
Within−cluster dispersion
12
14
16
18
20
22
24
Number of clusters (k)
Gap Statistic
Gap_k
0.46
0.48
0.50
0.52
Number of clusters (k)
