# Supplementary material for: Nurse shift patterns, staffing and their association with perceived workload: Sequence analysis of multicentre data
Source: Int J Nurs Stud Adv. 2025 Sep 12;9:100420. doi: 10.1016/j.ijnsa.2025.100420 (PMC12538485; doi:10.1016/j.ijnsa.2025.100420)
Supplement: Supplementary file 1 [file mmc1.zip › mmc1.zip!/Suppl_Material /Nurse_shift_workload_Supp_TableS1.docx]

**Table S1**

*Comparison between study sample and excluded sample*

|  | **Study sample (n=1,962)** | **Excluded sample due to missing data (n=501)** | ***p-*value** |
| --- | --- | --- | --- |
| Age (mean (SD)) | 35.5 (10.7) | 38.0 (11.4) | <0.001^t^ |
| Sex: female (%) | 88.8 | 85.4 | 0.050^c^ |
| Employment status: full-time (%) | 44.6 | 40.3 | 0.094^c^ |
| Last shift type: night (%) | 21.4 | 17.7 | 0.082^c^ |
| Last shift overtime: at least 0.5 hours (%) | 35.8 | 31.3 | 0.070^c^ |
| Last shift length in hours (mean (SD)) | 8.8 (1.2) | 8.9 (2.2) | 0.821^t^ |
| Overall perceived workload (mean (SD)) | 66.5 (17.2) | 63.9 (21.4) | 0.013^t^ |

*Note.* SD, standard deviation; t, *t*-test; c, chi-square test; n, sample size
