# Supplementary material for: Nurse shift patterns, staffing and their association with perceived workload: Sequence analysis of multicentre data
Source: Int J Nurs Stud Adv. 2025 Sep 12;9:100420. doi: 10.1016/j.ijnsa.2025.100420 (PMC12538485; doi:10.1016/j.ijnsa.2025.100420)
Supplement: Supplementary file 1 [file mmc1.zip › mmc1.zip!/Suppl_Material /Nurse_shift_workload_Supp_FigureS3.pdf]

$$
\begin{aligned}
\eta_{\text{perceived workload}} = {} & \beta_0 + \beta_1\text{Staffing individual nurse level} + \beta_2\text{Staffing unit} - \text{level} \\
& + \beta_3\text{Last shift type} + \beta_4\text{Last shift (hours)} + \beta_5\text{Overtime last shift} + \beta_6\text{Shift lenght system} \\
& + \beta_7\ \text{\%days worked overtime} + \beta_8\text{Clusters} + \beta_9\ \text{quick returns} + \beta_{10}\ \text{\% days worked} \\
& + \beta_{11}\text{age} + u_{unit} + u_{hospital} + \epsilon_i
\end{aligned}
$$
