# Supplementary material for: Nurse shift patterns, staffing and their association with perceived workload: Sequence analysis of multicentre data
Source: Int J Nurs Stud Adv. 2025 Sep 12;9:100420. doi: 10.1016/j.ijnsa.2025.100420 (PMC12538485; doi:10.1016/j.ijnsa.2025.100420)
Supplement: Supplementary file 1 [file mmc1.zip › mmc1.zip!/Suppl_Material /Nurse_shift_workload_Supp_FigureS2.pdf]

$$\eta_{\text{patient-to-nurse ratio}} = \beta_0 + \beta_1 \text{RNs} + \beta_2 \text{ Other staff} + \beta_3 \text{Total Patients} + \beta_4 \text{Last shift type} + \beta_5 \text{Patient acuity} + \beta_6 \text{Unit type} + u_{unit} + u_{hospital} + \epsilon_i$$
