# Supplementary material for: Nurse shift patterns, staffing and their association with perceived workload: Sequence analysis of multicentre data
Source: Int J Nurs Stud Adv. 2025 Sep 12;9:100420. doi: 10.1016/j.ijnsa.2025.100420 (PMC12538485; doi:10.1016/j.ijnsa.2025.100420)
Supplement: Supplementary file 1 [file mmc1.zip › mmc1.zip!/Suppl_Material /Nurse_shift_workload_Supp_FigureS7.pdf]

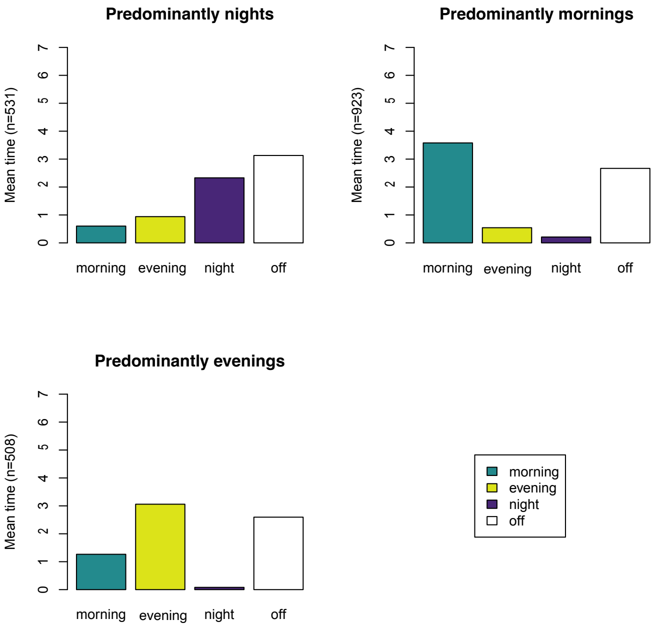

Predominantly nights
Mean time (n=531)
0
1
2
3
4
5
6
7
morning
evening
night
off
Predominantly mornings
Mean time (n=923)
0
1
2
3
4
5
6
7
morning
evening
night
off
Predominantly evenings
Mean time (n=508)
0
1
2
3
4
5
6
7
morning
evening
night
off
morning
evening
night
off
