# Supplementary material for: Nurse shift patterns, staffing and their association with perceived workload: Sequence analysis of multicentre data
Source: Int J Nurs Stud Adv. 2025 Sep 12;9:100420. doi: 10.1016/j.ijnsa.2025.100420 (PMC12538485; doi:10.1016/j.ijnsa.2025.100420)
Supplement: Supplementary file 1 [file mmc1.zip › mmc1.zip!/Suppl_Material /Nurse_shift_workload_Supp_FigureS5.pdf]

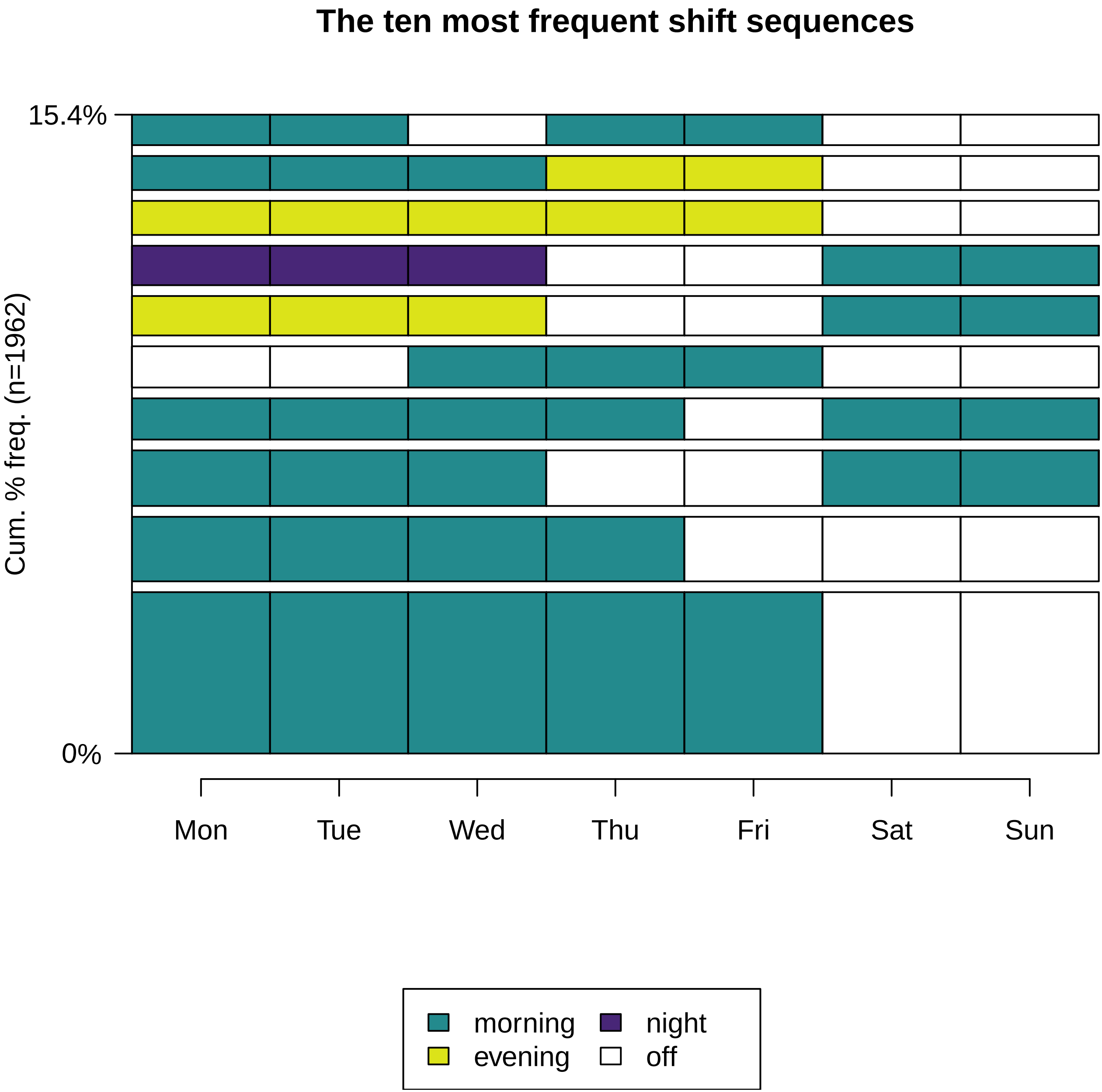

The ten most frequent shift sequences
15.4%
0%
Cum. % freq. (n=1962)
Mon
Tue
Wed
Thu
Fri
Sat
Sun
morning
evening
night
off
