# Supplementary material for: Nurse shift patterns, staffing and their association with perceived workload: Sequence analysis of multicentre data
Source: Int J Nurs Stud Adv. 2025 Sep 12;9:100420. doi: 10.1016/j.ijnsa.2025.100420 (PMC12538485; doi:10.1016/j.ijnsa.2025.100420)
Supplement: Supplementary file 1 [file mmc1.zip › mmc1.zip!/Suppl_Material /Nurse_shift_workload_Supp_FigureS4.pdf]

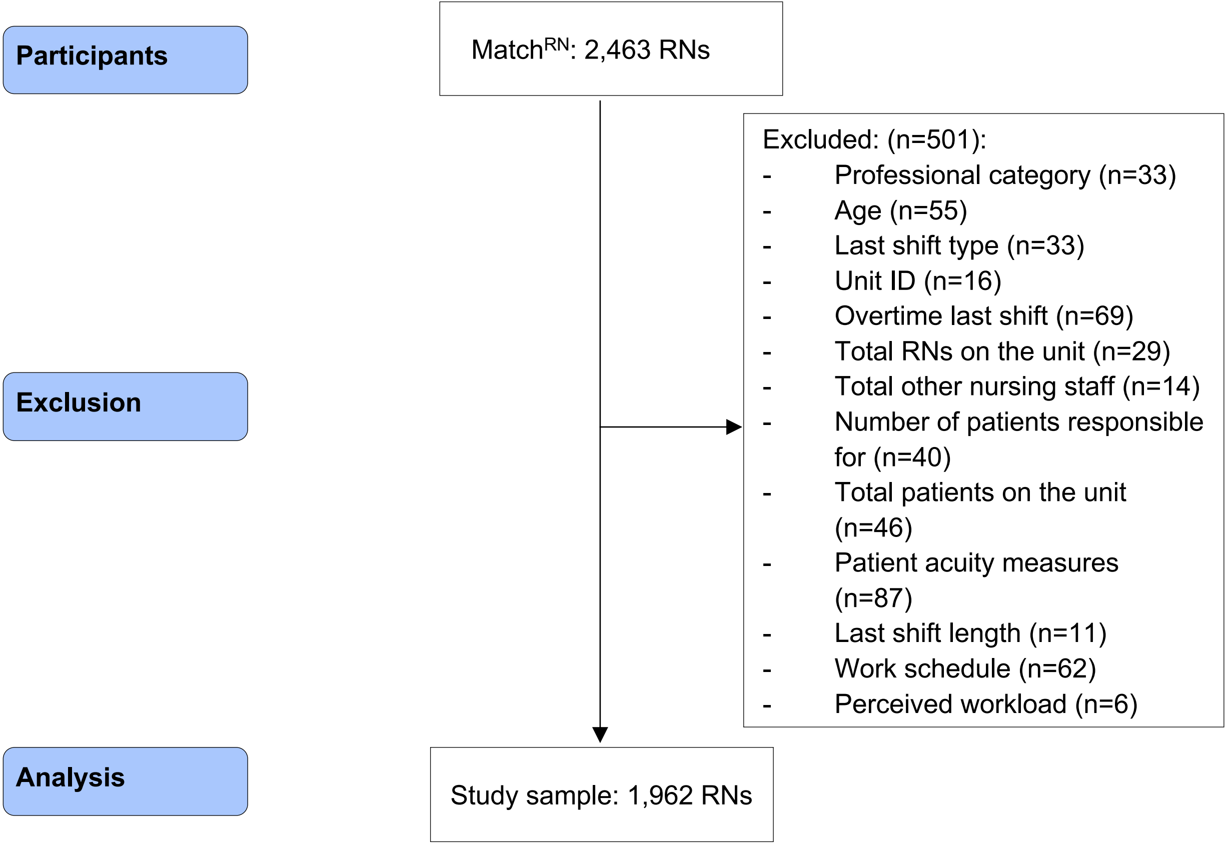
Participants
Exclusion
Analysis
Match^{RN}: 2,463 RNs
Excluded: (n=501):
- Professional category (n=33)
- Age (n=55)
- Last shift type (n=33)
- Unit ID (n=16)
- Overtime last shift (n=69)
- Total RNs on the unit (n=29)
- Total other nursing staff (n=14)
- Number of patients responsible for (n=40)
- Total patients on the unit (n=46)
- Patient acuity measures (n=87)
- Last shift length (n=11)
- Work schedule (n=62)
- Perceived workload (n=6)
Study sample: 1,962 RNs
