# Supplementary material for: Nurse shift patterns, staffing and their association with perceived workload: Sequence analysis of multicentre data
Source: Int J Nurs Stud Adv. 2025 Sep 12;9:100420. doi: 10.1016/j.ijnsa.2025.100420 (PMC12538485; doi:10.1016/j.ijnsa.2025.100420)
Supplement: Supplementary file 1 [file mmc1.zip › mmc1.zip!/Suppl_Material /Nurse_shift_workload_Supp_TableS3.docx]

**Table S3**

*Shift transitions – proportions*

| **Shifts** | [ -> morning] | [-> evening] | [-> night] | [ -> off] |
| --- | --- | --- | --- | --- |
| [ morning ->] | 0.64 | 0.08 | 0.02 | 0.26 |
| [ evening ->] | 0.04 | 0.59 | 0.05 | 0.32 |
| [ night ->] | 0.00 | 0.01 | 0.64 | 0.35 |
| [ off ->] | 0.23 | 0.13 | 0.05 | 0.59 |

*Note.* E.g. evening to morning transitions 0.04 = 4.0% of the transitions were quick returns*.*
