# Supplementary material for: Nurse shift patterns, staffing and their association with perceived workload: Sequence analysis of multicentre data
Source: Int J Nurs Stud Adv. 2025 Sep 12;9:100420. doi: 10.1016/j.ijnsa.2025.100420 (PMC12538485; doi:10.1016/j.ijnsa.2025.100420)
Supplement: Supplementary file 1 [file mmc1.zip › mmc1.zip!/Suppl_Material /Nurse_shift_workload_Supp_TableS2.docx]

**Table S2**

*Overtime characteristics*

| **Variables** | **n (%)** | **Mean (SD)** | **Median (IQR)** | **Range** |
| --- | --- | --- | --- | --- |
| Overtime - last shift (h) (only ≥ 0.5h) | 703 (35.8) | 0.85 (0.6) | 0.6 (0.5–1.0) | 0.5–6.1 |
| Overtime - previous seven days (h) | 592 (30.2) | 2.1 (1.6) | 1.8 (1.0–3.0) | 0.25–9.5 |
| Overtime - n° of days over the previous seven days |  | 2.2 (1.3) | 2.0 (1.0–3.0) | 1.0–6.0 |
| ≥5 | 34 (5.7) |  |  |  |
| 3-4 | 184 (31.1) |  |  |  |
| <3 | 374 (63.2) |  |  |  |

*Note.* SD, standard deviation; IQR, interquartile range; h, hours; n, sample size
