# Supplementary material for: Nurse shift patterns, staffing and their association with perceived workload: Sequence analysis of multicentre data
Source: Int J Nurs Stud Adv. 2025 Sep 12;9:100420. doi: 10.1016/j.ijnsa.2025.100420 (PMC12538485; doi:10.1016/j.ijnsa.2025.100420)
Supplement: Supplementary file 1 [file mmc1.zip › mmc1.zip!/Suppl_Material /Nurse_shift_workload_Supp_TableS5.docx]

**Table S5**

*Initial prediction model results (n=1,962)*

| **Variables** | **Estimates (**β**)** | **95% CI** | ***p*-value** |
| --- | --- | --- | --- |
| Intercept | 42.6 | 32.8–52.33 | <0.001 |
| Staffing individual level |  |  |  |
| O/E: low | 3.1 | 0.8–5.6 | 0.018 |
| O/E: medium | 2.1 | 0.4–4.1 | 0.040 |
| Last shift type: day | 3.8 | 1.7–5.7 | <0.001 |
| Last shift length | 1.1 | 0.1–2.0 | 0.026 |
| Twelve hours shifts: yes | 3.6 | –1.3–8.6 | 0.145 |
| Cluster: predominantly mornings^1^ | 0.2 | –1.7–2.1 | 0.866 |
| Cluster: predominantly evenings^1^ | 0.7 | –1.4–2.7 | 0.557 |
| Days worked overtime (%) | 3.9 | 1.3–6.3 | 0.002 |
| Overtime last shift: yes | 9.0 | 7.4–10.7 | <0.001 |
| Percentage of days worked | 6.5 | 2.6–10.3 | 0.001 |
| Quick returns: no | –1.4 | –5.0–2.3 | 0.469 |
| Age | 0.02 | -0.05–0.1 | 0.597 |
| **Random Effects** | **Variance (SD)** |  |  |
| Unit (n=158) | 22.4 (SD 4.7) |  |  |
| Hospital (n=26) | 14.9 (SD 3.8) |  |  |
| R^2^ marginal: 0.144 / R^2^ conditional: 0.269  Number of observations: 1,962; number of units: 158; number of hospitals: 26  AIC: 16289 | | | |

*Note.* Estimates are unstandardized; CI, confidence interval; SD, standard deviation, AIC, Akaike information criterion; R^2^, coefficient of multiple determination.
