# Supplementary material for: Nurse shift patterns, staffing and their association with perceived workload: Sequence analysis of multicentre data
Source: Int J Nurs Stud Adv. 2025 Sep 12;9:100420. doi: 10.1016/j.ijnsa.2025.100420 (PMC12538485; doi:10.1016/j.ijnsa.2025.100420)
Supplement: Supplementary file 1 [file mmc1.zip › mmc1.zip!/Suppl_Material /Nurse_shift_workload_Supp_TableS4.docx]

**Table S4**

*Staffing prediction model results (n=1,962)*

| **Variables** | **IRR** | **95% CI** | ***p*-value** |
| --- | --- | --- | --- |
| Intercept | 9.6 | 8.8–10.4 | <0.001 |
| Total n° of patients (unit) | 1.03 | 1.03–1.03 | <0.001 |
| Total n° of RNs (unit) | 0.81 | 0.80–0.83 | <0.001 |
| Total n° of other care staff (unit) | 0.99 | 0.97–1.00 | 0.325 |
| Last shift type: day^a^ | 0.75 | 0.72–0.79 | <0.001 |
| Patient acuity  (%) of patients responsible for who… |  |  |  |
| …required assistance with all ADL’s (%) | 0.99 | 0.99–0.99 | <0.001 |
| …required hourly or more monitoring/ treatments (%) | 0.99 | 0.99–0.99 | <0.001 |
| …were admitted or discharged (%) | 0.99 | 0.99–0.99 | 0.004 |
| Unit type^b^ |  |  |  |
| Medicine | 1.02 | 0.97–1.07 | 0.397 |
| Mixed | 1.04 | 0.97–1.11 | 0.208 |
| **Random Effects** | **Variance (SD)** |  |  |
| Unit (n=158) | 1.0 (SD 1.0) |  |  |
| Hospital (n=26) | 1.0 (SD 1.0) |  |  |
| R^2^ marginal: 0.536 / R^2^ conditional: 0.560  Number of observations: 1,962; number of units: 158; number of hospitals: 26  AIC: 8753 | | | |

*Note.* IRR, incidence rate ratio; SD, standard deviation; AIC, Akaike information criterion; n, sample size; R^2^, coefficient of multiple determination.

^a^Reference category: night

^b^Reference category: surgical
